# Uncovering Potential Roles of Differentially Expressed Genes, Upstream Regulators, and Canonical Pathways in Endometriosis Using an In Silico Genomics Approach

**DOI:** 10.3390/diagnostics10060416

**Published:** 2020-06-19

**Authors:** Zeenat Mirza, Umama A. Abdel-dayem

**Affiliations:** 1 King Fahd Medical Research Center, King Abdulaziz University, Jeddah 21589, Saudi Arabia; uabdelsalam@kau.edu.sa; 2 Department of Medical Lab Technology, Faculty of Applied Medical Sciences, King Abdulaziz University, Jeddah 21589, Saudi Arabia

**Keywords:** endometriosis, microarray, transcriptomics, biomarker, canonical pathways

## Abstract

Endometriosis is characterized by ectopic endometrial tissue implantation, mostly within the peritoneum, and affects women in their reproductive age. Studies have been done to clarify its etiology, but the precise molecular mechanisms and pathophysiology remain unclear. We downloaded genome-wide mRNA expression and clinicopathological data of endometriosis patients and controls from NCBI’s Gene Expression Omnibus, after a systematic search of multiple independent studies comprising 156 endometriosis patients and 118 controls to identify causative genes, risk factors, and potential diagnostic/therapeutic biomarkers. Comprehensive gene expression meta-analysis, pathway analysis, and gene ontology analysis was done using a bioinformatics-based approach. We identified 1590 unique differentially expressed genes (129 upregulated and 1461 downregulated) mapped by IPA as biologically relevant. The top upregulated genes were *FOS*, *EGR1*, *ZFP36*, *JUNB*, *APOD*, *CST1*, *GPX3*, and *PER1*, and the top downregulated ones were *DIO2*, *CPM*, *OLFM4*, *PALLD*, *BAG5*, *TOP2A*, *PKP4*, *CDC20B*, and *SNTN*. The most perturbed canonical pathways were mitotic roles of Polo-like kinase, role of Checkpoint kinase proteins in cell cycle checkpoint control, and ATM signaling. Protein–protein interaction analysis showed a strong network association among FOS, EGR1, ZFP36, and JUNB. These findings provide a thorough understanding of the molecular mechanism of endometriosis, identified biomarkers, and represent a step towards the future development of novel diagnostic and therapeutic options.

## 1. Introduction

Endometriosis is a painful gynecological ailment marked by the presence of endometrial tissue outside the uterine cavity, commonly involving the uterus, ovaries, fallopian tubes, and pelvic tissues [1]. It is a complex and chronic estrogen-dependent disorder, wherein abnormal growth of uterine-lining (endometrium) tissue occurs outside the uterus, which can lead to serious complications like diabetes, obesity, mood disorders, dysmenorrhea, chronic pelvic pain, or even fatal endometrial cancer and cardiovascular disorders if left untreated for long. The most common site of endometriosis is the Douglas pouch (rectovaginal region) of the pelvic peritoneum [2]. Common symptoms include agonizing abdominal pain, period cramps (dysmenorrhea), heavy periods, pain with bowel movements or urination, dyspareunia, and infertility [3].

Pelvic exams and sonography are done to visualize abnormalities, and laparoscopy is required for diagnosis as well as treatment. Modes of treatment are primarily hormonal suppression and ultrasonographically guided surgical/laparoscopic management, which only provide symptomatic relief and the condition can recur with time [4]. Unfortunately, invasive surgery and the lack of a disease biomarker presently causes a mean latency of 7–11 years from symptom onset to definitive diagnosis. This substantial lag possibly has negative consequences in terms of disease progression.

An estimated 176 million women are affected by it worldwide, and studies suggest that 10% of females of reproductive age suffer from this inflammatory disorder. The prevalence of endometriosis is 11.1% among Saudi Arabian women [5]. However, the condition is often underdiagnosed and undertreated. Future higher risk for endometrial polyps [6] and rare progression to endometriosis-associated adenocarcinoma exists in endometriosis patients. They also have a lifetime predisposition to clear-cell and endometrioid types of ovarian cancer [7], which are endometriosis-derived, and which are possibly associated with retrograde menstruation [8]. The identification of a sufficiently specific and sensitive marker for the non-surgical detection of endometriosis would promise early diagnosis and prevention of detrimental effects, underscoring the need for comprehensive research. The most extensively studied potential biomarker for endometriosis is cancer antigen 125, but its use as a sole diagnostic marker is impractical due to its low sensitivity [9].

Recent innovations of high-throughput transcriptomics-based genome-wide approaches have had a major impact on medical research [10], thereby aiding in clinical classification and treatment predictions [10,11,12]. Understanding the genetic basis of the pathophysiology of endometriosis is important to explain the strong genetic association with heritability, estimated at around 50% [13]. Dysregulation of several genes has been implicated in the etiology of this ectopic condition.

In a menstrual cycle, endometrium undergoes transition from estrogen-dominant proliferative (follicular) phases (early-proliferative (EP), mid-proliferative (MP), and late-proliferative (LP)) to progesterone-dominant secretory (luteal) phases ((early-secretory (ES), mid-secretory (MS), and late-secretory (LS)), followed by the menstrual phase. A distinct differential transcriptional profile exists for each endometrial cycle phase [14,15]. As the uterine linings in endometriosis patients have altered transcriptomic profiles, molecular classification is needed for disease identification and staging [16]. Previous works have focused on expression profiling of different stages of the endometrial cycle in small groups. Herein, we integrated data to conduct comprehensive differential transcriptional profiling of a large cohort in order to identify differentially expressed genes, upstream regulators, and perturbed canonical pathways that could possibly be used in future to identify novel potential biomarkers and therapeutic targets for endometriosis.

### Etiology

Decrease in age of menarche, fewer pregnancies, less breast feeding, and increase in maternal age at first birth all cause an overall increase in the number of ovulations and menstruations within a reproductive lifespan. These changes are associated strongly with the risk of endometriosis development and tend to be more pronounced during the decade of highest risk for endometriosis, i.e., 25–35 years of age [17]. Estrogen dependence, immune modulation, and certain environmental pollutants, mostly dioxins and polychlorinated biphenyls, perhaps contribute to the disease’s pathobiology [18]. Immunological or hormonal dysfunction make some women predisposed to endometriosis. Higher macrophage activation and humoral immune responsiveness with reduced cell-mediated immunity, with weakened T-cell and NK-cell responses, are seen in women suffering from endometriosis. Humoral autoantibodies against endometrial and ovarian tissue have been detected in endometriosis patient sera [19].

The pathogenesis of endometriosis has been speculated to result from aberrant angiogenesis that occurs in the eutopic endometrium with retrograde menstruation—“Sampson’s hypothesis” [2]. Factors that increase the rate of retrograde menstruation, such as congenital outflow tract obstructions, might also predispose to endometriosis. Detailed understanding on the basis of gene expression studies is lacking, and findings are often inconsistent or even contradictory.

## 2. Materials and Methods

### 2.1. Data Retrieval and Sample Description

Our approach was the integration of publicly available gene expression data generated by different microarray platforms. We first retrieved whole-transcript array datasets (.CEL files) along with provided clinical details of endometriosis patients dated up to 30 March 2020 from the Gene Expression Omnibus (GEO, NCBI) databank, a public domain hosting high-throughput genomic data. The present study included following expression data series with GEO accession numbers GSE7846, GSE7305, GSE6364, GSE4888, GSE51981, GSE31683, and GSE25628 and their sample information to compare the transcriptomic status of affected and control patients (Table 1). The GSE51981 dataset has a total of 148 endometrial samples from patients with ages ranging from 20–50 years. It includes samples from women in different menstrual cycle phases, including endometriosis with severe pelvic pain/infertility (*n* = 77) and normal without endometriosis (*n* = 71). Normal women with uterine fibroids, adenomyosis, or pelvic organ prolapse were further grouped as normal with uterine/pelvic pathology (*n* = 37), and others as normal without uterine pathology (*n* = 34). The GSE7846 dataset includes five arrays for human endometrial endothelial cells (HEECs) derived from eutopic endometria of patients with endometriosis, and five from patients without endometriosis (controls). GSE7305 includes expression profiles of 10 each of normal and diseased cases.

### 2.2. Gene Expression Analysis

To generate expression profiles of endometriosis samples, .CEL files were imported to Partek Genomics Suite, version 7.0 (Partek Inc., St. Louis, MO, USA) followed by log-transformation and normalization of the robust background-adjusted array dataset. Principal component analysis (PCA) was done on high-dimensional data to assess quality and overall variance in gene expression of individuals among sample groups. Analysis of variance (ANOVA) was employed to create a list of differentially expressed genes (DEGs) with a cut-off *p*-value of ≤ 0.05 and fold change of ± 2. Hierarchical clustering was done to reveal the pattern of most differentially expressed (up- and downregulated) genes across samples.

### 2.3. Gene Ontology, Pathway, and Upstream Regulators Analysis

The identified statistically significant DEGs with corresponding probe sets ID, p-value, fold-change values, and other relevant data were uploaded into the Ingenuity Pathways Analysis (IPA, QIAGEN’s Ingenuity Systems, Redwood City, CA, USA) software for molecular network and canonical pathway analysis to define interaction amongst the differentially regulated genes using functional algorithms. The Benjamini–Hochberg method was used to adjust p-values for canonical pathways, and *p*-values below 0.01 and Altman z-scores of ± 2 were considered significant. Positive and negative values of z-score represent activation and inhibition of dysregulated canonical pathways. Gene ontology study was done to functionally categorize endometriosis-significant genes. All endometriosis-associated DEGs were imported to figuratively represent all identified connections and potential relationships among them, in order to identify significant pathways leading to endometriosis initiation and progression.

### 2.4. Protein–Protein Interaction Analysis

To check the interactions at the protein level, the STRING v11.0 database (http://string-db.org) was used to search for possible physical and functional associations among proteins encoded by the top DEGs (including both up- and downregulated) for a better understanding of disease pathobiology [20]. This prediction gives a visual idea about the possible interconnections between the proteins involved in a specific disease network.

## 3. Results

### Differentially Expressed Genes from Meta-Analysis

Integration of the seven GEO data series included in present study comprised a total of 156 endometriosis patients and 118 controls. Data were merged before analysis as all had used the same GPL570 platform, except GSE25628 which used the GPL571 platform (Table 1).

**Figure 1:**
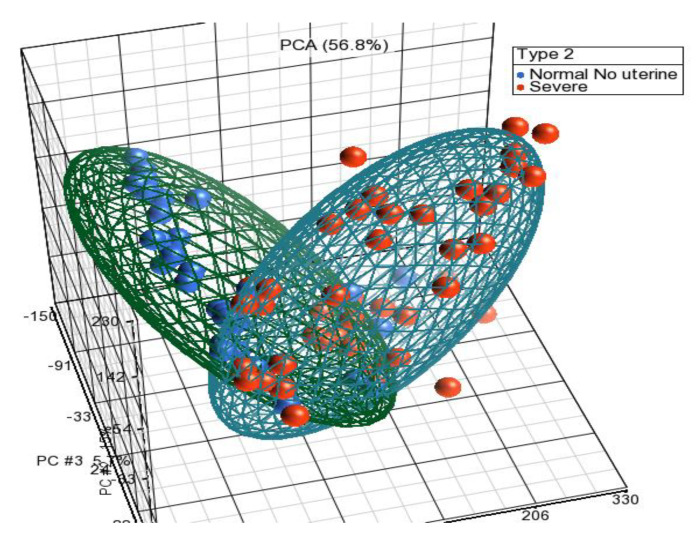
PCA showing two clear distinct clusters for severe endometriosis cases and normal healthy controls without uterine pathology.

**Figure 2:**
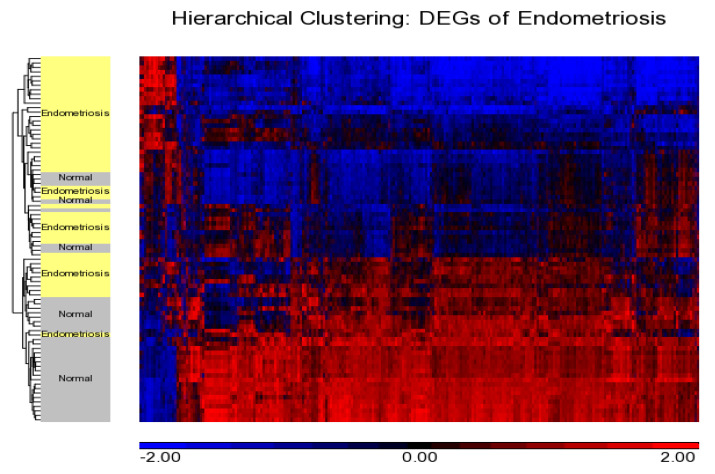
Hierarchical clustering showing distribution of DEGs and cases. Downregulated and upregulated genes are shown in blue and red, respectively, showing a distinct pattern with a majority of genes found to be downregulated in endometriosis.

Principal component analysis showed the grouping of the samples in three-dimensional space as per their whole-genome expression patterns, where each circle represents an individual (Figure 1). Comparing endometriosis with normal non-endometriosis tissue without any pelvic/uterine pathology resulted in the detection of 1590 differentially expressed genes (129 upregulated and 1461 downregulated). The top upregulated genes, including *FOS*, *EGR1*, *ZFP36*, *JUNB*, *APOD*, *CST1*, *GPX3*, and *PER1*, are shown in Table 2 and the top downregulated genes, including *DIO2*, *CPM*, *OLFM4*, *PALLD*, *BAG5*, *TOP2A*, *PKP4*, *CDC20B*, and *SNTN*, are shown in Table 3. Hierarchical clustering of DEGs showed a clear difference in expression pattern of genes between endometriosis cases and controls (Figure 2). Disease and functional annotation of DEGs broadly predicted endometrial adenocarcinoma. However, DEGs like *FOS*, *EGR1*, *ZFP36*, *JUNB*, *GPX3*, *PAEP*, *DUSP1*, *MT1M*, *COL6A1*, *NR4A1*, *TGFB1*, *CITED2*, *IL2RG*, *ACKR1*, *JUN*, *PTGER3*, *COL6A2*, *PGR*, *PLK2*, *PLA2G4A*, *FBN1*, *MPPED2*, *EZR*, *MMP11*, *GALNT4*, *PTEN*, *PIK3CA*, *CREB1*, *ERBIN*, *DNMT3A*, *REL*, *SDC2*, *ZNF25*, *ITGA6*, *GUCY1A2*, *PDGFD*, *OVGP1*, *ITGB1*, *APOBEC3B*, *OLFM1*, *NRIP1*, *MEF2A*, *CNTN1*, *BUB1B*, *MEST*, *KIF20A*, *RRM1*, *ANK3*, and *CCNA2* showed significant association with endometriosis (*p*-value = 0.0006).

**Figure 3:**
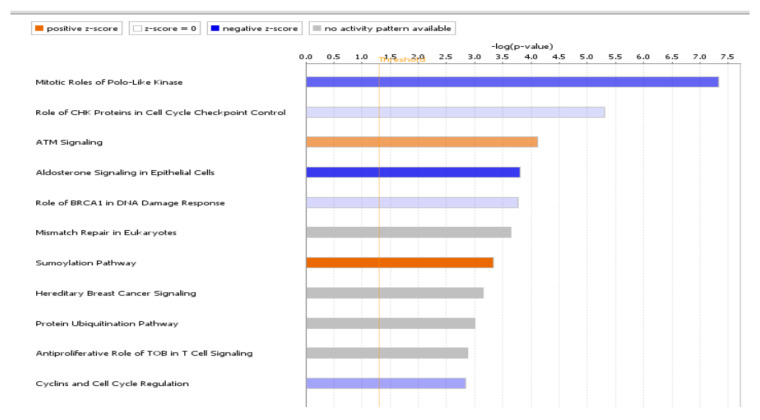
Significant pathways identified by IPA. The top 11 altered canonical pathways predicted from DEGs of endometriosis. Negative and positive z-scores, colored in shades of blue and red, represent inhibition and activation of pathways, respectively.

**Figure 4:**
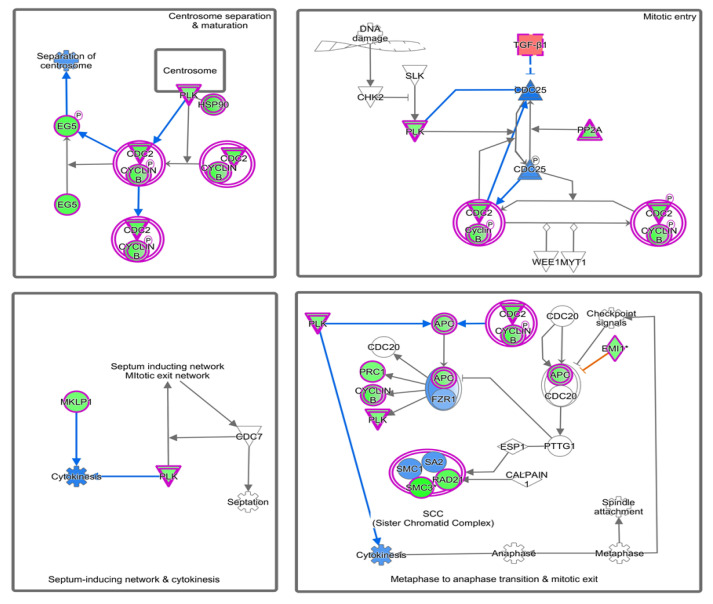
Significant inhibition of mitotic polo-like kinase pathway predicted by IPA. Pathway genes were overlaid to the DEGs; upregulated (*TGFB1*, *CDC25*, *FZR1*) genes are shown in red/pink, while downregulated genes (*PRC1*, *PLK*, *Cyclin B1*, *SMC3*) are shown in green.

**Figure 5:**
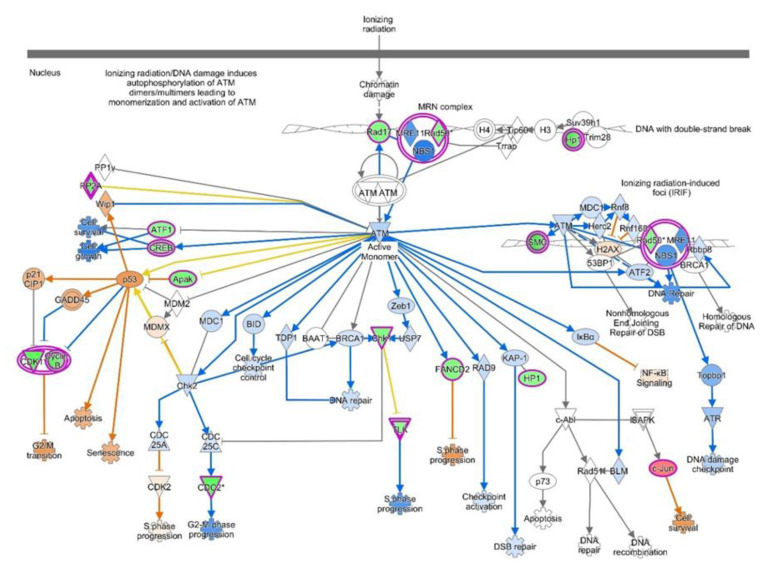
Activation of ATM signaling predicted by IPA-based pathway analysis. Pathway genes were overlaid to the DEGs, with upregulated ones shown in shades of red and downregulated ones shown in green.

**Figure 6:**
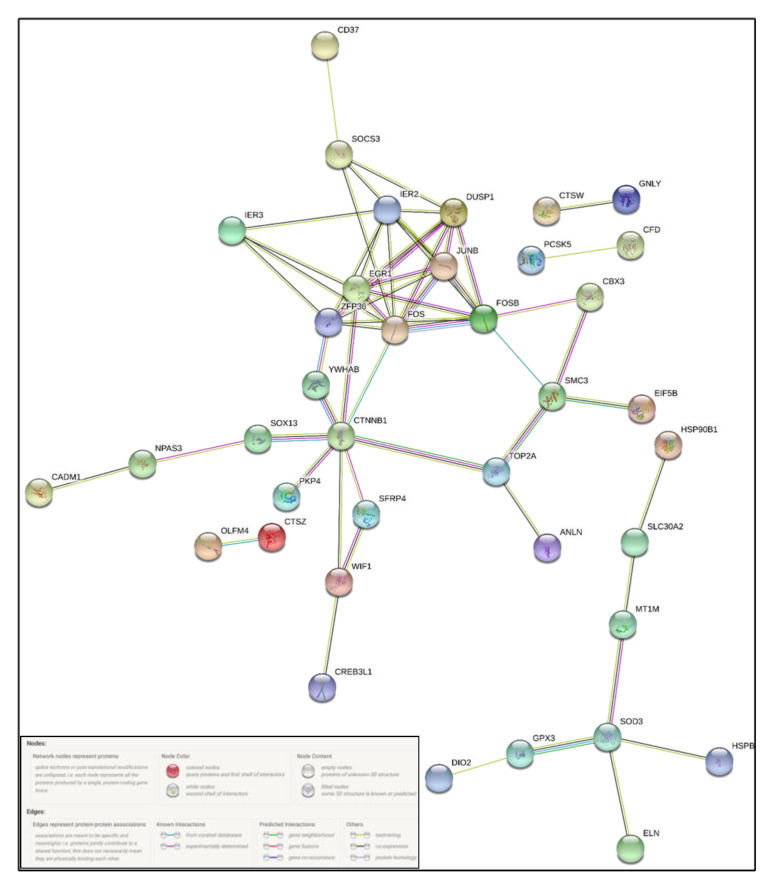
STRING-based protein–protein interaction (PPI) network of selected differentially expressed genes, showing predicted interconnectivities.

Ingenuity pathway analysis for the DEGs of endometriosis revealed altered canonical pathways that were either activated or inhibited (Figure 3, Table 4). Mitotic roles of polo-like kinase (z-score −2.71), aldosterone signaling in epithelial cells (z-score −3.464), and role of CHK proteins in cell cycle checkpoint control (z-score −0.632) were found to be inhibited while ATM signaling (z-score + 1.698) and SUMOylation pathways (z-score + 2.668) were activated (Figure 4, Figure 5). IPA predicted the activation status of upstream regulators among identified DEGs of endometriosis. REL (transcription factor, z-score −4.13, Pval 0.0002), CTNNB1 (transcription factor, z-score −3.2, Pval 0.01), PGR (ligand-dependent nuclear receptor, z-score −2.2, Pval 0.0005), and VCAN (proteoglycan, z-score −2.6, Pval 0.02) were the top inhibited upstream regulators (Table 5). We also used a biological database, STRING, to predict functional associations and interaction between the proteins encoded by the identified significant DEGs (top up- and downregulated ones), and the results are shown in Figure 6. The network indicated a strong interplay of various proteins and their specific involvement in endometriosis.

## 4. Discussion

Endometriosis, a growth/deposition of endometrial tissue at extra-uterine sites, affects around 10% of reproductive women. In addition to abnormal reproductive physiological problems, cases are increasing drastically due to adverse consequences of treatment with oral contraceptives, GnRH agonists, synthetic progestins, and aromatase inhibitors (letrozole) to prevent the menstrual cycle and/or pregnancy [1,21]. Understanding the molecular etiology of origin and progression of endometriosis is necessary to explore therapeutic options and provide better treatment. We therefore conducted transcriptomic meta-analysis to identify endometriosis-associated significant DEGs and essential pathological pathways.

Combining multiple studies has always been challenging, as different studies use varied protocols, platforms, and analysis methods. We used raw data (.CEL) files to integrate multiple data series to get a bigger cohort and analyzed the data. We identified transcriptomic signatures of endometriosis and evaluated the roles of specific genes, upstream regulators, and dysregulated pathways. Our results provide some insight into the molecular mechanisms underlying endometriosis pathogenesis. Pathogenic genes and pathways may serve as novel targets for diagnostic and prognostic biomarkers and potential therapies for endometriosis. In the present study, we had a long list of genes and pathways, but have restricted our discussion to the most prominent genes and pathways.

### 4.1. Molecular Etiology of Endometriosis

Retrograde or “reverse” menstruation has been suggested as an initial cause of endometriosis, where menstrual blood is thrown back into pelvic cavity outside the uterus, instead of flowing out of the cervix. This endometrial tissue growth out of the uterus is the result of an estrogen-dependent hormonal local imbalance. Higher prevalence has been also seen in women with immune disorders (like rheumatoid arthritis, multiple sclerosis, systemic lupus erythematosus, and hypo- or hyperthyroidism) [17]. Recently, a small-molecule agonist G-1 (Tespria) against the G-protein-coupled estrogen receptor also showed reduction in endometrial growth [22].

Unusual transformation of certain abdominal wall cells into endometrial cells has been reported in some women [23] and, interestingly, it is believed that during embryonic development, the same cells are responsible for the growth of female reproductive organs. Researchers also think that pelvic inflammation, damage, or infection of cells that line the pelvis like a prior caesarean surgery can also trigger endometriosis [23,24,25]. The exact pathogenesis still remains uncertain. We therefore conducted a transcriptomics study in order to understand the genetic factors that allow cells to grow as endometrial tissue outside the uterus.

In our results, we found high expression of early and immediate early-response genes such as FBJ murine osteosarcoma viral oncogene homolog or *Fos proto-Oncogene* (*FOS*), *FosB Proto-Oncogene (FOSB), Early Growth Response 1 (EGR1), ZFP36 Ring Finger Protein (ZFP36), Immediate Early Response 2 (IER2), Immediate Early Response 3 (IER3), Jun B Proto-Oncogene (JUNB)*, and *Transcription Factor SOX-13 (SOX 13)*. The majority of these are DNA-binding proteins that act as transcriptional factors. Some others, like *Dual specificity protein phosphatase 1 (DUSP1)* and *Receptor-type tyrosine-protein phosphatase O (PTPRO),* possess phosphatase activity.

c-Fos is the transcription factor of the Fos family, including *FosB*, *Fra-1*, and *Fra-2* [26]. It is an immediate early-response gene involved in cell proliferation and differentiation of normal tissue after extracellular stress stimuli. Its deregulation has been linked to oncogenic transformation and tumor progression. *FOS* plays a significant role in endometrial cells’ proliferation and its overexpression is associated with a poor prognosis of endometrial carcinoma [27]. Fos and Jun family proteins form a heterodimer complex of AP-1 transcription factor, shown to be involved in endometrial carcinogenesis [28]. Upstream regulator analysis revealed genes such as *REL*, *CTNNB1*, *PGR*, and *VCAN* by analyzing linkage to DEGs that were experimentally shown to affect gene expression [29]. All upstream regulators were inhibited.

**REL:***REL* (*V-Rel Avian Reticuloendotheliosis Viral Oncogene Homolog*) encodes for the proto-oncogene c-Rel protein, a transcription factor of the NF-κB family that regulates genes involved in B- and T-cell differentiation, immune response, survival, apoptosis, proliferation, and oncogenic processes, including endometrial carcinogenesis [30,31].

**CTNNB1:***CTNNB1* (*Catenin β1*) codes for a protein that regulates and coordinates cell–cell adhesion, embryonic development, epithelial–mesenchymal transition, and gene transcription. It is an integral part of the canonical Wnt pathway. Aberrant Wnt/β-catenin signaling pathway function is allied with loose cytoskeleton organization and cell-to-cell contacts of epithelial cells, along with a high motility of mesenchymal cells to promote invasiveness and fibrosis. This might lead to multiple cancers, including endometrial cancer [32,33,34,35,36]. Targeting the Wnt/β-catenin signaling was shown to avert fibrogenesis in a xenograft endometriosis mice model [35].

**VCAN:***VCAN* (*Versican*) codes for four extracellular matrix isoforms like large chondroitin sulfate proteoglycan in different tissues and organs that regulate cell adhesion, proliferation, migration, and survival [2]. Higher expression of *VCAN* has been reported in angiogenesis, tumor growth, cancer relapse, and inflammatory lung disorders [37,38,39]. Significantly high expression of *VCAN* was also reported in the mid-secretory phase of endometrial epithelial cells after combination estrogen/progesterone treatment. The V1 isoform of *VCAN* was recently reported to the facilitate development of endometrial receptivity and human embryo implantation [40]. Higher expression of *VCAN* is connected with pathogenesis of peritoneal endometriosis and seems to be an indicator of poor prognosis endometrial cancer [2,41].

Alteration in expression of *HOXB4* [42], apelin peptide [43], interleukin 18 [44], estrogen and progesterone receptors [45], integrin β3 and osteopontin (*OPN*) [46], microRNA-29c, and FKBP4 [47] have been reported. Varied expression levels of metastasis-inducing proteins (*S100P*, *S100A4*, *OPN*, and anterior gradient homologue 2 (*AGR2*)) have been shown to enhance pathogenesis by increasing endometrial cell invasiveness and establishing endometriotic ectopic deposits after retrograde menstruation [48].

Aromatase activates estrogen biosynthesis locally from androgens, thereby sequentially stimulating a positive feedback cycle of prostaglandin E2 production by upregulating cyclooxygenase-2 (*COX-2*). Because of lack of aromatase (estrogen synthase) in the normal endometrium, androgens cannot be converted into estrogen [49]. In contrast, numerous studies have described aberrantly high expression of aromatase in eutopic and ectopic endometrium [17]. Increased *COX-2* expression in the stromal cells and aberrant aromatase overexpression in eutopic endometrium have both been indicated as potential therapeutic biomarkers, and therefore, their specific inhibitors are being increasingly employed for therapeutic management [50]. A probable connection of Krüppel-like Factor 9 (*KLF9*) dysregulation has been suggested in both pregnancy failure and endometrial pathogenesis [51]. The progesterone resistance and subsequent infertility seen in endometriosis seems to have an association with *KLF9*, a progesterone-receptor-interacting protein, as mice null for Klf9 are sub-fertile. It is implicated that deficiency of *KLF9* contributes to progesterone resistance of eutopic endometrium in patients [52] and exhibits simultaneous abrogation of Hedgehog-, Notch-, and steroid-receptor-regulated networks [53].

Based on serum proteomic differential expression, a possible biomarker panel comprising zinc-alpha-2-glycoprotein, albumin, and complement C3 has been proposed for effective and non-invasive diagnosis of endometriosis [54]. Importantly, the three markers were independent from the endometriosis stage and cycle phase. Brain Derived Neurotrophic Factor (*BDNF*) has been identified as a potential peripheral early diagnostic marker, as its mean plasma concentrations were twice as high in endometriosis cases in contrast to asymptomatic or healthy controls [9]. Based on this, a nano-chip-based electrochemical detection technique was developed. The only limitation to this is its non-specificity, as the variations in *BDNF* expression have been reported in numerous unconnected pathologies [55].

### 4.2. Canonical Pathways Involved in Endometriosis

Molecular pathway analysis revealed a couple of significantly altered canonical pathways for DEGs of endometriosis. Herein, we discuss the role of key pathways like Mitotic roles of polo-like kinase, Role of CHK proteins in cell cycle checkpoint control, Aldosterone signaling in epithelial cells, and ATM Signaling in endometriosis progression.

**Mitotic Roles of Polo-Like Kinase Pathway:** The Polo-like kinase (Plks) is a member of the serine/threonine protein kinase (*PLK1-5*) family that regulates the mitotic checkpoint during M phase of cell division. Plks can act either as oncogene or tumor suppressor, and has been found to be overexpressed in different cancer types including endometrial [56] and ovarian [57] cancers. Because of its direct association with increased cellular proliferation and poor prognosis, it is considered a bona fide cancer biomarker [58,59]. Direct association of Plks expression with serum estrogen (ovarian hormone) levels and abnormal regulation of ectopic endometrial cell proliferation strongly suggest its role in the pathogenesis of endometriosis [60]. Plks inhibitors such as volasertib and rigosertib are in advanced stage of clinical trials and might be used for endometriosis treatment [61].

**Role of CHK Proteins in Cell Cycle Checkpoint Control Pathway:** Activation of cell cycle checkpoint kinases including Chk1 and Chk2 are an instant response to repair any type of DNA damage [62]. In response to DNA damage, this signaling pathway temporarily delays cell cycle progression, allowing time for DNA repair, or triggers programmed cell death. Activated ATM kinase phosphorylates Chk2 which phosphorylates *CDC25C* to block the progression from G2 to M phase. Chk2 also phosphorylates p53, attenuating p53 binding to *MDM2* and activating p21/WAF1 to arrest the G1 phase of the cell cycle. *Rad3*-dependent activation of Chk1 phosphorylates *CDC25A* and *CDC2* to inhibit their activity to block G2–M transition. Overall, CHK protein signaling depends on the type of stress and extent of DNA damage and is involved in endometrial cancer [63].

Cisplatin exerts an anticancer effect by activating DNA-damage-response genes Chk1/2, which generates both survival (repair) and apoptotic signals that lead to cell death. Cisplatin-resistant cells have dominant repair signaling that allows cells to survive. Chk1/2 inhibitor AZD7762 has been shown to overcome cisplatin resistance in endometriosis-associated ovarian cancer by reducing repair signaling [64].

**ATM Signaling Pathway:** Ataxia telangiectasia mutated (*ATM*) gene codes for serine/threonine protein kinase and participates in cell division and DNA repair. DNA damage induces autophosphorylation of ATM which activates DNA repair enzymes by phosphorylating Chk1/2 to fix the broken strands [65]. Efficient cross-talk between ATR-Chk1 and ATM-Chk2 leads to repair of damaged DNA strands which helps to maintain the cell’s genomic stability and integrity [66]. The ATM signaling pathway, because of its central role in cell division and DNA repair, has been a focus of cancer research, especially endometrial cancer, for exploring novel molecular therapies targeting ATM pathways [67].

**Aldosterone Signaling in Epithelial Cells Pathway:** Aldosterone is a mineralocorticoid steroid hormone produced by the adrenal cortex. Aldosterone signaling primarily controls blood pressure and inflammation by regulating its target genes (*FKBP5*, *IGF1*, *KRAS*, *PKCε*, *NCOA1*, *NCOR1*, *NEDD4L*, *SGK*, and *MR/NR3C2* as per RGD, https://rgd.mcw.edu [68] and IPA). Recent studies have shown the possible involvement of aldosterone in multiple gynecological problems and inflammatory disorders [69]. There is a well-established association of endometriosis with intraperitoneal inflammation diseases like atherosclerosis and hypertension, and also with autoimmune diseases like diabetes, hypothyroidism, and cancer [9]. A metabolomics-based study revealed high aldosterone levels in endometriosis patients with infertility [70].

### 4.3. Future Directions

The strength of present work lies in the inclusion of multiple endometriosis-related expression datasets in order to understand endometriosis at the molecular level. However, the absence of a validation study was its limitation. In future, we plan to conduct RT-PCR-based validation studies for differentially expressed genes on endometriosis samples collected from the Jeddah region. Further cell cultures and animal models could be used to assess the effect of activated/suppressed genes on molecular pathways and disease phenotypes for potential clinical translation. Virtual screening of potential lead compounds against identified therapeutic biomarkers for rational drug design will be done. This could facilitate imminent tailor-made personalized therapies.

## 5. Conclusions

Endometriosis is an estrogen-dependent, progesterone-resistant, inflammatory multifactorial gynecological disorder. Identification of distinct molecular signatures and potential therapeutic molecules corresponding to endometriosis is needed for better diagnosis. The present microarray-based genomics and molecular pathway analysis method helped to establish a better understanding of endometriosis at the molecular level, as multiple expression datasets were integrated to determine differentially expressed genes and identify canonical molecular pathways related to endometriosis in a broad way. The study identified alterations of gene expression and molecular signaling, including aldosterone signaling, that result in the hormonal imbalances and pathogenesis of endometriosis. An anti-inflammatory diet and increased levels of antioxidants and phytonutrients can be recommended to patients to reverse inflammation and oxidative damage, while also supporting healthy hormone balance.

## Figures and Tables

**Table 1:** **Gene expression Omnibus** (GEO) data series, platform, and sample description of endometrium-based expression studies (total 156 endometriosis patients + 118 normal cases).

GEO Data Series	Total No. of Cases (Diseased + Normal)	Platform	Sample Description
GSE 7846	10 (5 + 5)	GPL570	Endometriosis (*n* = 5), Normal (*n* = 5)
GSE 7305	20 (10 +10)	GPL570	Endometriosis (*n* = 10), Normal (*n* = 10)
GSE 6364	37 (21 + 16)	GPL570	Proliferative (*n* = 6), Proliferative normal (*n* = 5); Early-secretory (*n* = 6), Early-secretory normal (*n* = 3); Mid-secretory (*n* = 9), Mid-secretory normal (*n* = 8)
GSE 4888	27 (21 + 6)	GPL570	Proliferative (*n* = 4), Early-secretory (*n* = 3), Mid-secretory (*n* = 8), Late-secretory (*n* = 6), Ambiguous histology (*n* = 6)
GSE 51981	148 (77 + 71)	GPL570	Severe endometriosis (*n* = 48), Mild endometriosis (*n* = 29), Normal without pelvic/uterine pathology (*n* = 34), Normal with pelvic/uterine pathology (*n* = 37)
GSE 31683	10 (6 + 4)	GPL570	*KLF9* silenced (*n* = 2), *PGR* silenced (*n* = 2), Both *KLF9* and *PGR* silenced (*n* = 2), Normal (*n* = 4)
GSE 25628	22 (16 + 6)	GPL571	Eutopic (*n* = 9), Ectopic (*n* = 7), Normal (*n* = 6)

**Table 2:** Top overexpressed/upregulated differentially expressed genes in endometriosis.

Gene Symbol	Gene Name	*p*-Value	Fold-Change
*FOS*	*FBJ murine osteosarcoma viral oncogene homolog*	3.02 × 10^−13^	9.94558
*EGR1*	*early growth response 1*	7.67 × 10^−17^	7.95823
*FOSB*	*FBJ murine osteosarcoma viral oncogene homolog B*	1.87 × 10^−10^	7.14284
*ZFP36*	*ZFP36 ring finger protein*	5.23 × 10^−14^	4.01565
*JUNB*	*jun B proto-oncogene*	8.28 × 10^−15^	4.01404
*APOD*	*apolipoprotein D*	1.21 × 10^−7^	3.7376
*CST1*	*cystatin SN*	2.52 × 10^−5^	3.42216
*GPX3*	*glutathione peroxidase 3*	0.0057	3.34348
*PER1*	*period circadian clock 1*	6.14 × 10^−13^	3.23599
*CTSW*	*cathepsin W*	8.08 × 10^−7^	3.22223
*SOCS3*	*suppressor of cytokine signaling 3*	4.99 × 10^−5^	3.11348
*CFD*	*complement factor D (adipsin)*	0.000993	2.99141
*HSPB6*	*heat shock protein, alpha-crystallin-related, B6*	9.31 × 10^−8^	2.98362
*LEFTY1*	*left-right determination factor 1*	0.004712	2.89285
*PAEP*	*progestagen-associated endometrial protein*	0.029113	2.88223
*DUSP1*	*dual specificity phosphatase 1*	1.48 × 10^−7^	2.88219
*GNLY*	*Granulysin*	1.52 × 10^−5^	2.83639
*EPHX1*	*epoxide hydrolase 1, microsomal (xenobiotic)*	6.69 × 10^−10^	2.78618
*MT1M*	*metallothionein 1M*	0.011648	2.76574
*CLEC3B///EXOSC7*	*C-type lectin domain family 3, member B///exosome component 7*	7.13 × 10^−5^	2.74002
*GNLY*	*Granulysin*	1.61 × 10^−5^	2.73433
*TPSAB1///TPSB2*	*tryptase alpha/beta 1///tryptase beta 2 (gene/pseudogene)*	1.20 x10^−6^	2.73381
*IER2*	*immediate early response 2*	4.65 × 10^−14^	2.67138
*PTPRO*	*protein tyrosine phosphatase, receptor type, O*	2.70 × 10^−14^	2.66719
*ELN*	*Elastin*	2.26 × 10^−7^	2.6637
*IER3*	*immediate early response 3*	5.70 × 10^−5^	2.61981
*SOX13*	*SRY box 13*	4.59 × 10^−9^	2.60666
*SOD3*	*superoxide dismutase 3, extracellular*	2.00 × 10^−9^	2.60457
*SLC30A2*	*solute carrier family 30 (zinc transporter), member 2*	0.002566	2.59508
*AQP3*	*aquaporin 3 (Gill blood group)*	0.000604	2.58706
*HSPB6*	*heat shock protein, alpha-crystallin-related, B6*	2.81 × 10^−6^	2.57421
*CD37*	*CD37 molecule*	3.56 × 10^−8^	2.48625
*IRX3*	*iroquois homeobox 3*	0.00585	2.48441
*CREB3L1*	*cAMP responsive element binding protein 3-like 1*	1.53 × 10^−7^	2.48023

**Table 3:** The top downregulated differentially expressed genes in meta-analysis of endometriosis.

Gene Symbol	Gene Title	*p*-Value	Fold-Change
*DIO2*	*deiodinase, iodothyronine, type II*	6.84 × 10^−12^	−5.38638
*CPM*	*carboxypeptidase M*	1.52 × 10^−7^	−5.20763
*OLFM4*	*olfactomedin 4*	1.22 × 10^−6^	−4.77761
*PALLD*	*palladin, cytoskeletal associated protein*	1.20 × 10^−13^	−4.47775
*BAG5*	*BCL2-associated athanogene 5*	1.62 × 10^−8^	−4.38993
*TOP2A*	*topoisomerase (DNA) II alpha*	4.50 × 10^−8^	−4.22713
*PKP4*	*plakophilin 4*	5.35 × 10^−19^	−3.97475
*CDC20B*	*cell division cycle 20B*	1.36 × 10^−6^	−3.96043
*SNTN*	*sentan, cilia apical structure protein*	5.43 × 10^−12^	−3.95899
*SET*	*SET nuclear proto-oncogene*	5.39 × 10^−11^	−3.90488
*CRISPLD1*	*cysteine-rich secretory protein LCCL domain containing 1*	5.12 × 10^−7^	−3.85982
*NPAS3*	*neuronal PAS domain protein 3*	1.57 × 10^−6^	−3.83298
*CADM1*	*cell adhesion molecule 1*	4.63 × 10^−15^	−3.78248
*SMC3*	*structural maintenance of chromosomes 3*	2.59 × 10^−16^	−3.7407
*SFRP4*	*secreted frizzled-related protein 4*	0.000481	−3.72407
*ANK2*	*ankyrin 2, neuronal*	2.00 × 10^−7^	−3.71876
*ANLN*	*anillin actin binding protein*	4.54 × 10^−8^	−3.70838
*WIF1*	*WNT inhibitory factor 1*	1.73 × 10^−6^	−3.65345
*MMP26*	*matrix metallopeptidase 26*	0.002107	−3.63998
*PCSK5*	*proprotein convertase subtilisin/kexin type 5*	7.33 × 10^−8^	−3.63558
*OXR1*	*oxidation resistance 1*	1.62 × 10^−13^	−3.61905
*CTNNB1*	*catenin (cadherin-associated protein), beta 1*	6.39 × 10^−12^	−3.55057
*PCSK5*	*proprotein convertase subtilisin/kexin type 5*	1.69 × 10^−8^	−3.53439
*EIF5B*	*eukaryotic translation initiation factor 5B*	1.60 × 10^−8^	−3.52153
*HSP90B1*	*heat shock protein 90kDa beta (Grp94), member 1*	2.11 × 10^−9^	−3.49988
*PCYOX1*	*prenylcysteine oxidase 1*	3.06 × 10^−11^	−3.47374
*MIB1*	*mindbomb E3 ubiquitin protein ligase 1*	1.96 × 10^−9^	−3.46544
*OSBPL1A*	*oxysterol binding protein-like 1A*	2.08 × 10^−14^	−3.46429
*CBX3*	*chromobox homolog 3*	2.18 × 10^−12^	−3.45307
*TCAF1*	*TRPM8 channel-associated factor 1*	1.87 × 10^−13^	−3.42665
*KMO*	*kynurenine 3-monooxygenase (kynurenine 3-hydroxylase)*	2.06 × 10^−5^	−3.42531
*CTSZ*	*cathepsin Z*	3.75 × 10^−13^	−3.40201
*KMO*	*kynurenine 3-monooxygenase (kynurenine 3-hydroxylase)*	2.13 × 10^−5^	−3.38857
*PCSK5*	*proprotein convertase subtilisin/kexin type 5*	5.06 × 10^−8^	−3.38299
*YWHAB*	*tyrosine 3-monooxygenase/tryptophan 5-monooxygenase activation protein, beta*	2.64 × 10^−13^	−3.35541
*NMT2*	*N-myristoyltransferase 2*	5.60 × 10^−12^	-3.34186
*CADM1*	*cell adhesion molecule 1*	3.18 × 10^−9^	−3.3281
*CEP57*	*centrosomal protein 57kDa*	3.21 × 10^−15^	−3.28628

**Table 4:** Significant canonical pathways based on DEGs of endometriosis. Positive and negative z- scores indicate overall activation and inhibition status of pathways, respectively.

Ingenuity Canonical Pathways	−log (*p*-Value)	Ratio	Predicted z-Score	Molecules
Mitotic Roles of Polo-Like Kinase	7.34	0.333	−2.714 (Inhibited)	*ANAPC4, CCNB1, CCNB2, CDC23, CDC27, CDK1, FBXO5, HSP90AA1, HSP90AB1, HSP90B1, KIF11, KIF23, PLK2, PPM1L, PPP2R2C, PPP2R5C, PPP2R5E, PRC1, RAD21, SMC3, TGFB1*
ATM Signaling	4.11	0.219	1.698 (Activated)	*ATF1, CBX1, CBX3, CBX5, CCNB1, CCNB2, CDK1, CHEK1, CREB1, FANCD2, JUN, PPM1L, PPP2R2C, PPP2R5C, PPP2R5E, RAD17, RAD50, SMC2, SMC3, TLK1, ZNF420*
Aldosterone Signaling in Epithelial Cells	3.8	0.183	−3.464 (Inhibited)	*DNAJA1, DNAJB14, DNAJC10, DNAJC27, DNAJC3, DNAJC9, DUSP1, HSP90AA1, HSP90AB1, HSP90B1, HSPA4, HSPA9, HSPB6, HSPD1, HSPH1, ITPR2, PDIA3, PDPK1, PIK3C2A, PIK3CA, PIK3R3, PIP5K1B, PLCB1, PRKCI, PRKD3, SACS, SCNN1G*
Role of CHK Proteins in Cell Cycle Checkpoint Control	5.3	0.298	−0.632	*ATMIN, CDK1, CHEK1, E2F7, E2F8, PCNA, PPM1L, PPP2R2C, PPP2R5C, PPP2R5E, RAD17, RAD50, RFC3, RFC4, RFC5, RPA1, TLK1*
SUMOylation Pathway	3.34	0.198	2.668 (Activated)	*DNMT3A, EP300, FOS, HDAC2, JUN, MYB, PCNA, PIAS1, RFC3, RFC4, RFC5, RHOB, RHOBTB1, RHOQ, RHOT1, RND3, RPA1, SERBP1, SMAD4, UBA2*
Role of BRCA1 in DNA Damage Response	3.77	0.225	−0.707	*ABRAXAS1, ATF1, BRCC3, BRD7, BRIP1, CHEK1, E2F7, E2F8, FANCD2, FANCL, MSH2, MSH6, RAD50, RFC3, RFC4, RFC5, RPA1, SMARCC1*
Cyclins and Cell Cycle Regulation	2.83	0.2	−1.604	*CCNA1, CCNA2, CCNB1, CCNB2, CCNE2, CDK1, CDK6, E2F7, E2F8, HDAC2, PPM1L, PPP2R2C, PPP2R5C, PPP2R5E, SKP2, TGFB1*
PI3K/AKT Signaling	2.7	0.171	−1.091	*CTNNB1, EIF4E, HSP90AA1, HSP90AB1, HSP90B1, INPP5F, ITGB1, JAK1, PDPK1, PIK3CA, PIK3R3, PPM1L, PPP2R2C, PPP2R5C, PPP2R5E, PTEN, RASD1, RHEB, RPS6KB1, SFN, SYNJ2, YWHAB*

**Table 5:** Most significant upstream regulators and target molecules in endometriosis dataset.

Upstream Regulator	Molecule Type	Predicted Activation z-score	*p*-Value of Overlap	Target Molecules in Dataset
*REL*	transcription regulator	−4.137 (Inhibited)	0.00021	*AGA, AGPS, ANLN, APP, ARFGAP3, ATXN1, BCL3, CAMK2D, CCNY, CDC6*
*CTNNB1*	transcription regulator	−3.208 (Inhibited)	0.0104	*AKAP13, ALDH1A1, APOD, APP, ARFGAP3, ARMH4, AURKA, CADM1, CALM1, CCL3*
*PGR*	ligand-dependent nuclear receptor	−2.237 (Inhibited)	0.00051	*ABCG2, ACOX1, AHCYL1, AK3, AKAP13, ATP1B1, ATXN1, BUB1, CA12, CCNB1*
*VCAN*	Proteoglycan	−2.625 (Inhibited)	0.024	*COMP, CPE, ELN, IFI44L, IFIT1, ITGB1, MYH10, PCSK5, PENK, PLA2G2A*
*ACTL6A*	Other	−2.236 (Inhibited)	0.093	*CCNA2, CCNB1, CCNB2, CCNE2, SFN*
*DUSP1*	phosphatase	−2.155 (Inhibited)	1	*CMPK2, DUSP1, IER3, IFIT1, IFIT3, JUN, PTEN, ZFP36*
*HELLS*	Enzyme	−2 (Inhibited)	0.0024	*CCNA2, CCNB1, CDC6, HSPD1, PCNA, SLC44A1*
*RASSF8*	Other	−2 (Inhibited)	0.0016	*ENPP5, MRPL30, NEDD9, POSTN*
*TCF4*	transcription regulator	−1.912	0.040	*CCNA2, CCNB2, CDK1, CEP55, E2F8, FOS, HMGB2, HMMR, HSP90B1, IFI16*
*IGF2R*	transmembrane receptor	−2 (Inhibited)	0.0080	*ENPP5, MRPL30, NEDD9, POSTN*
*TGFB1*	growth factor	−1.276	0.00087	*ABCG2, ACKR1, ADAM12, ALDH5A1, APP, ARHGAP19, ASPM, ATG12, ATXN1, BCL3*
*HSF2*	transcription regulator	−1.408	0.026	*CCT2, HSBP1, HSPA4, HSPH1, JUN, PSMA5, TCP1*
*EDN3*	other	0.816	0.0024	*CDH2, CTNNB1, EGR1, FOS, ITGB1, LAMA1*
*FOS*	transcription regulator	0.917	0.0020	*ACOX1, ADAM12, AGPS, ANK3, AQP3, ATP2C1, CADM1, CALU, CAMK2D, CAT*
*RTN4*	other	1.51	0.0041	*APP, CFL1, IMPACT, JUN, JUND, LAP3, MAP2, RHOB, RTN4, YWHAB*
*ZFP36*	transcription regulator	1.873	0.00015	*CCNE2, CDC6, CENPA, CLCN3, CLMP, CTSS, E2F8, FOS, IER3, JUN*
*EPHB1*	kinase	1.98	0.0052	*EGR1, FOS, JUN, JUNB*
